# The genetically encoded tool set for investigating cAMP: more than the sum of its parts

**DOI:** 10.3389/fphar.2015.00164

**Published:** 2015-08-06

**Authors:** Neha Patel, Matthew G. Gold

**Affiliations:** Department of Neuroscience, Physiology and Pharmacology, University College LondonLondon, UK

**Keywords:** cAMP, PKA, AKAP, adenylyl cyclase, phosphodiesterase

## Abstract

Intracellular fluctuations of the second messenger cyclic AMP (cAMP) are regulated with spatial and temporal precision. This regulation is supported by the sophisticated arrangement of cyclases, phosphodiesterases, anchoring proteins, and receptors for cAMP. Discovery of these nuances to cAMP signaling has been facilitated by the development of genetically encodable tools for monitoring and manipulating cAMP and the proteins that support cAMP signaling. In this review, we discuss the state-of-the-art in development of different genetically encoded tools for sensing cAMP and the activity of its primary intracellular receptor protein kinase A (PKA). We introduce sequences for encoding adenylyl cyclases that enable cAMP levels to be artificially elevated within cells. We chart the evolution of sequences for selectively modifying protein–protein interactions that support cAMP signaling, and for driving cAMP sensors and manipulators to different subcellular locations. Importantly, these different genetically encoded tools can be applied synergistically, and we highlight notable instances that take advantage of this property. Finally, we consider prospects for extending the utility of the tool set to support further insights into the role of cAMP in health and disease.

## Introduction

The discovery that some hormones elevate the second messenger cyclic AMP (cAMP) without triggering canonical effects such as increased phosphorylase activity (Keely, 1979; Hayes et al., 1980) indicated that cAMP may be compartmentalized in cells (Edwards et al., 2012). Studies of cAMP compartmentalization rely upon methods to sense and manipulate signaling enzyme activities in space and time (Scott and Pawson, 2009). The first sensor of intracellular cAMP (‘FlCRhR’) was constructed by fluorescein-labeling PKA catalytic subunits and rhodamine-labeling the regulatory subunits of the tetrameric kinase (Adams et al., 1991). Release of the catalytic subunits upon binding of cAMP to the regulatory subunits can be detected as a reduction in Forster resonance energy transfer (FRET; Adams et al., 1991). The FlCRhR probe was revolutionary as it enabled the first visualizations of cAMP compartmentalization (Bacskai et al., 1993; Hempel et al., 1996). However, the sensor must be microinjected since its fluorescent labels are not genetically encoded. This technical hurdle has limited the influence of the FlCRhR probe.

The subsequent development of genetically encoded sensors of cAMP (Zaccolo et al., 2000) and PKA activity (Zhang et al., 2001), in tandem with methods for manipulating both the enzyme that synthesizes cAMP – adenylyl cyclase (AC) – and molecular interactions that support cAMP signaling, has initiated a new wave of discoveries concerning cAMP signaling (Edwards et al., 2012). Studies with these novel tools have helped to establish the necessity for targeting PKA to its substrates by anchoring to A-kinase anchoring proteins (AKAPs; Rosenmund et al., 1994; Zhang et al., 2001; Di Benedetto et al., 2008). The tools have been applied to reveal a small nuclear population of anchored PKA (Sample et al., 2012). Furthermore, they have illuminated how PKA interacts spatiotemporally with phosphodiesterase (PDE) enzymes that degrade cAMP (Willoughby et al., 2006), with the exchange-protein activated by cyclic AMP (Epac; Dodge-Kafka et al., 2005), and with Ca^2+^ signals (Cooper and Tabbasum, 2014). This genetically encoded tool set has also been applied to establish a role for localized cAMP signaling in diseases (Gold et al., 2013b) such as heart failure (Nikolaev et al., 2010), muscular dystrophy (Roder et al., 2009), diabetes (Zhang et al., 2005), breast cancer (Hansen et al., 2009), and adrenal Cushing’s syndrome (Beuschlein et al., 2014).

In this review, we introduce genetically encoded sensors of cAMP and PKA activity, before discussing tools based upon ACs. We describe sequences that are available for targeting such tool proteins to specific sub-cellular locations and for modifying protein interactions involving cAMP signaling proteins. Importantly, further functionality can arise when the different classes of tools are combined, and we highlight studies that exploit this kind of synergy. Finally, we consider how the tool set might be extended and combined in novel ways to enable further advances in the understanding of cAMP signaling.

## Genetically Encoded Sensors for Monitoring cAMP Dynamics

There are three types of cAMP receptors in higher organisms: PKAs, Epacs, and cyclic-nucleotide-gated channels (CNGCs). Genetically encoded sensors are available that derive from each class of endogenous cAMP receptor (Willoughby and Cooper, 2008). Sensors derived from PKAs and Epacs rely upon fusions to variants of green fluorescent protein (GFP) that undergo decreased FRET upon elevation of cAMP. Sensors derived from CNGCs can also employ measurements using patch clamp electrophysiology to detect changes in conductance upon binding of cAMP to the channel. The first genetically encoded FRET-based cAMP sensor was constructed by fusing an improved GFP to the C-terminus of the PKA catalytic subunit and a blue GFP variant (EBFP) to the C-terminus of type-II regulatory (RII) PKA subunits (Zaccolo et al., 2000). Respective substitution of EBFP and GFP with cyan and yellow fluorescent proteins yielded an improved sensor that is less prone to photobleaching (Zaccolo and Pozzan, 2002). This allowed real time imaging of cAMP fluctuations in cardiomyocytes in response to the β-adrenergic agonist norepinephrine or the broad spectrum PDE inhibitor isobutyl-methyl-xanthine (IBMX), and helped to establish that type IV PDEs are the critical PDE class for degrading cAMP following activation with norepinephrine in cardiomyocytes (Mongillo et al., 2004).

**FIGURE 1:**
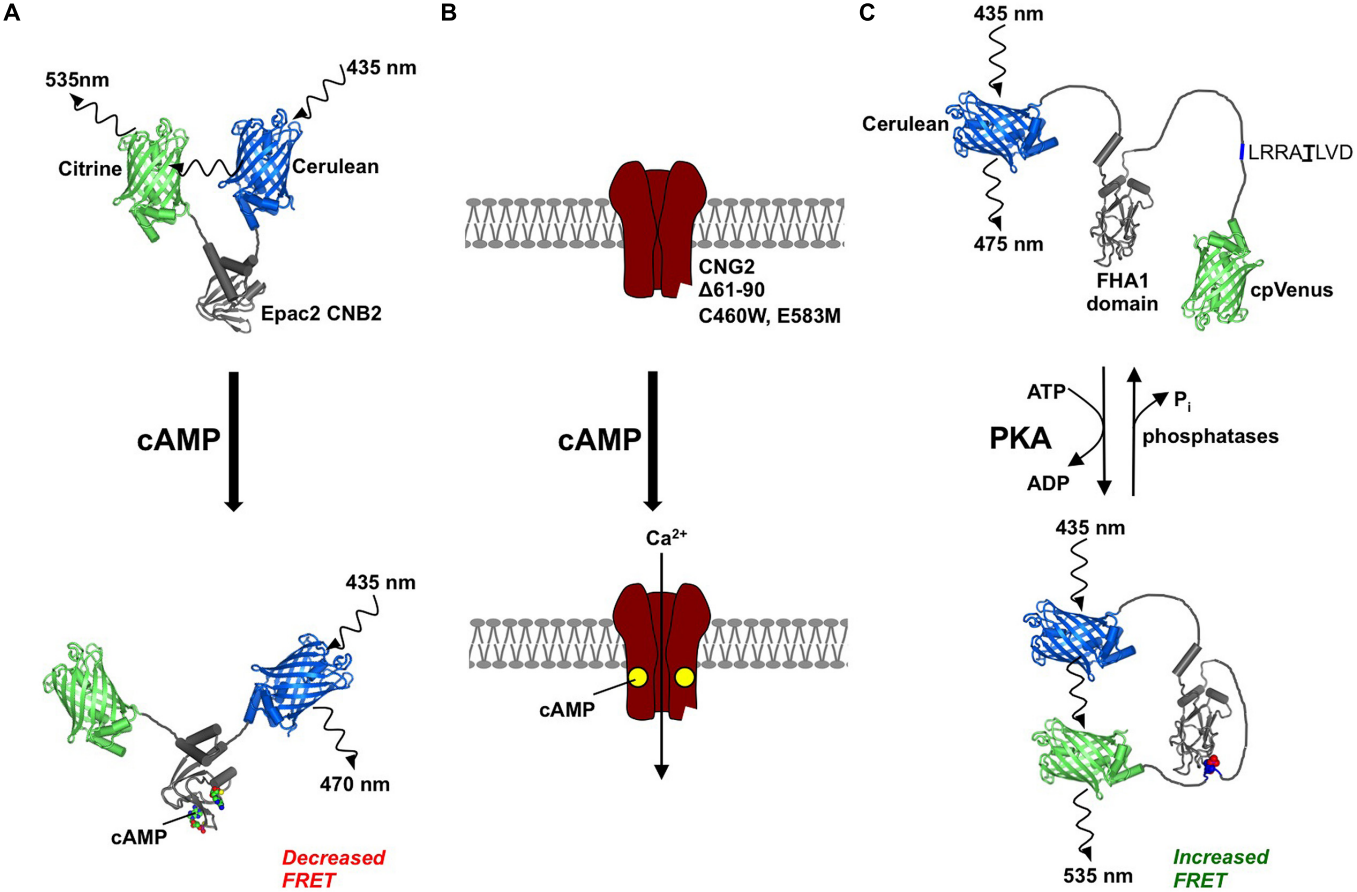
**Tools for monitoring cyclic AMP (cAMP) signaling.** Two cAMP sensors and an A-kinase activity reporter are illustrated. **(A)** Cartoon showing mechanism for cAMP sensing by ‘Ci/C-Epac2-camps,’ which is based on the second cAMP binding domain of Epac2. Elevation of cAMP leads to separation of citrine and cerulean fluorescent proteins that can be detected as a decrease in FRET **(B)** cAMP sensor based on cyclic nucleotide-gated channel 2 (CNG2), which is modified with removal of residues 61–90 to prevent Ca^2+^/calmodulin regulation, and the substitutions C460W/E583M for high affinity and specificity for cAMP. Elevation of cAMP leads to increased flux of Ca^2+^ which can be detected using Ca^2+^ dyes or by measuring current across the membrane. **(C)** AKAR4 contains a central FHA1 domain. PKA phosphorylation of threonine within the motif ‘LRRATLVD’ leads to association of phosphothreonine with FHA1, which can be detected as an increase in FRET between the terminal fluorescent proteins.

The discovery of Epac1 and Epac2 (de Rooij et al., 1998; Kawasaki et al., 1998) opened the door for development of unimolecular cAMP sensors based upon these cAMP-dependent GTPase activators (DiPilato et al., 2004; Nikolaev et al., 2004; Ponsioen et al., 2004). Unimolecular sensors based on Epacs exhibit higher FRET efficiency (increase in FRET signal from maximal to minimal cAMP) of ∼20–30% compared to ∼8% for multimolecular PKA-based sensors. Epac probes also show better temporal resolution (Nikolaev et al., 2004), and there are no concerns regarding balancing expression of the donor and acceptor fluorophores in unimolecular probes. For these reasons, Epac probes are now the most popular option for sensing cAMP fluctuations. The latest optimized Epac-based probes include pH-insensitive Ci/Ce Epac2-camps (Everett and Cooper, 2013) (**Figure 1A**) and the Epac1-based probes ICUE3 (DiPilato and Zhang, 2009) and ^T^Epac^VV^ (Klarenbeek et al., 2011; Li et al., 2015) although the original probes exhibit good FRET efficiency and are still popular. CNGC-based cAMP sensors excel in temporal resolution of cAMP fluctuations (Rich et al., 2000; Fagan et al., 2001). The first example of this approach exploited rat CNG2 expression in human embryonic kidney-293 (HEK-293) cells (Rich et al., 2000). Measurement of Ca^2+^ flux through the channels as a proxy for cAMP elevation using electrophysiology supported the existence of cellular cAMP microdomains. Mutations can be incorporated into the CNG2 to tailor it for sensing cAMP: C460W improves cAMP sensitivity, E583M improves cAMP specificity over cGMP, and removal of residues 61-90 abrogates channel regulation by Ca^2+^/calmodulin (Rich et al., 2001) (**Figure 1B**). A C460W/E583M double CNG2 mutant was used as a sensor to reveal that both G protein coupled receptor kinases (GRKs) and PKA stimulate PDE degradation of cAMP following β2-AR simulation of HEK-293 cells (Xin et al., 2008). A common way to apply CNG2 is to combine expression of the channel with the Ca^2+^ dye Fura-2, allowing measurement of Ca^2+^ influx by imaging rather than electrophysiology (Fagan et al., 2001; Rich et al., 2001, 2007; Rochais et al., 2004). For example, the E583M CNG2 variant was applied in this way to establish the necessity of PKA anchoring for negative feedback through PKA activation of type IV PDEs (Willoughby et al., 2006). CNGCs can also be adapted as FRET sensors (Nikolaev et al., 2006). The hyperpolarization-activated CNG2 (HCN2) has a higher sensitivity than CNG2, and exhibits as wide a dynamic range as a cAMP FRET sensor when YFP and CFP are fused either side of a single HCN2 cAMP binding domain (Nikolaev et al., 2006).

Genetically encoded cAMP probes have been applied to investigate different facets of cAMP signaling. For example, they have helped build upon initial observations of cAMP oscillations in Brooker (1973) and Gorbunova and Spitzer (2002). Epac-based probes demonstrate that cAMP oscillations can be evoked in cell lines including MIN6 cells (Landa et al., 2005) and HEK293 cells (Willoughby and Cooper, 2006). cAMP oscillations have also been monitored in β-cells using evanescent-wave-microscopy in combination with fluorescently labeled RII-CAAX and C subunits (Dyachok et al., 2008). This approach shows that Ca^2+^ amplifies but is not essential for glucose-induced cAMP oscillations in β-cells (Dyachok et al., 2008). Ca^2+^ typically oscillates in tandem with cAMP, and a related area of focus has been the basis of interplay between cAMP and Ca^2+^ signals at the level of signaling proteins. Epac probes reveal that distinct pools of cAMP center on specific isoforms of AC (Wachten et al., 2010), with the Ca^2+^-activated cyclase AC8 occupying a prominent role in linking Ca^2+^ signals to localized cAMP elevation (Willoughby et al., 2010; Ayling et al., 2012).

A key advantage of genetically encoding sensors is that transgenic animals expressing the sensors can be generated. Imaging of cAMP fluctuations has been achieved in pancreatic islets (Kim et al., 2008) using genetically engineered mice that selectively express a PKA-based cAMP sensor in pancreatic islets. This approach was exploited to show that that glucose triggers cAMP elevation independent of Ca^2+^ (Kim et al., 2008). Another study monitored cAMP changes upon activation of either β1 or β2 adrenergic receptors within small sarcolemmal areas by employing adrenergic receptor knockout mice transgenically expressing Epac2-camps (Nikolaev et al., 2010). This study revealed that β2 adrenergic receptors are restricted to deep transverse tubules (Nikolaev et al., 2010). Development of transgenic fruit flies expressing cAMP sensors (Lissandron et al., 2007) has also proved to be useful. In particular, *Drosophila* expressing Epac1-camps with an upstream activating sequence for GAL4 (Shafer et al., 2008) have enabled detailed investigation of how neuropeptides including pigment dispersing factor modulate cAMP in neurons during circadian rhythms (Duvall and Taghert, 2012; Pirez et al., 2013; Vecsey et al., 2014; Yao and Shafer, 2014). In sum, an impressive array of sensors and delivery options are now available for monitoring intracellular cAMP fluctuations.

## Fluorescence-Based Sensors for Monitoring PKA Activity

One potential limitation of cAMP sensors is that they may not reflect cAMP receptor activation if the receptors and active cyclase are not co-localized. For PKA, genetically encoded A-kinase activity reporters (AKARs) may be utilized to monitor kinase activity more directly (Mehta and Zhang, 2011). The first AKAR was constructed by placing YFP and CFP either side of a PKA consensus phosphorylation sequence derived from Kemptide and the phospho-serine/threonine-binding protein 14-3-3τ (Zhang et al., 2001). Phosphorylation at the PKA consensus sequence causes association of the central elements that may be detected as a concomitant increase in FRET (Zhang et al., 2001). A second-generation sensor, AKAR2, incorporates a Forkhead-associated (FHA) domain in tandem with a lower affinity PKA recognition site. This modification improves the reversibility of the reporter (Zhang et al., 2005). This reporter was first applied to investigate interaction of insulin and isoproterenol stimulation of adipocytes. Using AKAR2, the authors found that chronic insulin treatment delayed PKA activation following addition of low concentrations of the β-AR agonist isoproterenol (Zhang et al., 2005). Conversely, PKA response to stimulation with either forskolin or caged cAMP was not affected by prior chronic insulin treatment. This suggested that insulin reduces PKA localization to a pool of cAMP associated with β-ARs. This notion was corroborated by anti-β-AR immunoprecipitation experiments that revealed decreased interaction of PKA RII subunits with β-ARs following dual treatment with insulin and isoproterenol (Zhang et al., 2005). This detail might have been missed if a cAMP sensor had been employed rather than AKAR2. Systematic improvement of AKAR reporters is ongoing (Liu et al., 2011; Oldach and Zhang, 2014). The latest reporter, AKAR4, features the fluorescent protein variants Cerulean and cpVenus (Depry et al., 2011) (**Figure 1C**).

A-kinase activity reporters have also been widely applied. For example, they have been used to validate stapled PKA anchoring disruptor peptides (Wang et al., 2014), and to confirm that Leu206Arg substitution in Cα subunits (associated with Adrenal Cushing’s Syndrome) leads to constitutive kinase activation (Beuschlein et al., 2014). They are often utilized in parallel with cAMP sensors, for example, in imaging fluctuations of cAMP and PKA activity induced by neural activity in retinal cells (Dunn et al., 2006); to investigate cAMP/PKA dynamics at the centrosome (Terrin et al., 2012); and in the study of neurite outgrowth including a forskolin-coated glass bead contact procedure for cultured hippocampal neurons (Shelly et al., 2010). As with genetically encoded cAMP sensors, it is possible to express AKARs in transgenic animals. For example, PKA activity dynamics have been imaged in *Drosophila* expressing AKAR2 (Gervasi et al., 2010). In this study, Kamioka et al. (2012) performed crosses with learning and memory deficient mutant fly lines to establish that the AC Rutabaga acts as a coincidence detector during aversive and appetitive learning (Gervasi et al., 2010). AKAR-expressing mice have also been developed, and exploited to image real-time PKA activity in mouse epidermis and small intestine (Kamioka et al., 2012). These whole-organism studies underline the benefits of genetically encoding reporters in comparison to techniques that rely upon microinjection (Adams et al., 1991).

## Tools for Manipulating Adenylyl Cyclase Activity

The ability to control cAMP elevations with spatiotemporal precision can help to reveal how cAMP signaling is organized in time and space (Scott and Pawson, 2009). In analogous fashion to the discovery of light-activated channelrhodopsins for artificially depolarizing cells (Nagel et al., 2003), photo-active adenylyl cyclases (PACs) have been identified in photo-sensitive microbes (Iseki et al., 2002; Ryu et al., 2010; Stierl et al., 2011). Advantages of genetically encoded PACs over cAMP uncaging approaches (Ponsioen et al., 2004; Saucerman et al., 2006) include the ability to deliver into whole animals, and the option to localize the PAC within cells by fusion to subcellular targeting sequences. The first PAC to be characterized and utilized was discovered in *Euglena.* This unicellular flagellate relies on a PAC in photophobic behavior (Iseki et al., 2002). *Euglena* PAC comprises two subunits, PACα and PACβ. Each subunit consists of two blue light receptor using flavin adenine nucleotide (BLUF) domains paired with two AC domains. Activation of the BLUF domains with blue light leads to a conformational change that activates the AC domains. Both PAC subunits respond to blue light with a maximal increase in AC activity of ∼80-fold. The PACα subunit is more active than PACβ in both light and dark conditions (Iseki et al., 2002), so applications of *Euglena* PAC have utilized the α subunit (Bucher and Buchner, 2009; Bellmann et al., 2010; Weissenberger et al., 2011).

**FIGURE 2:**
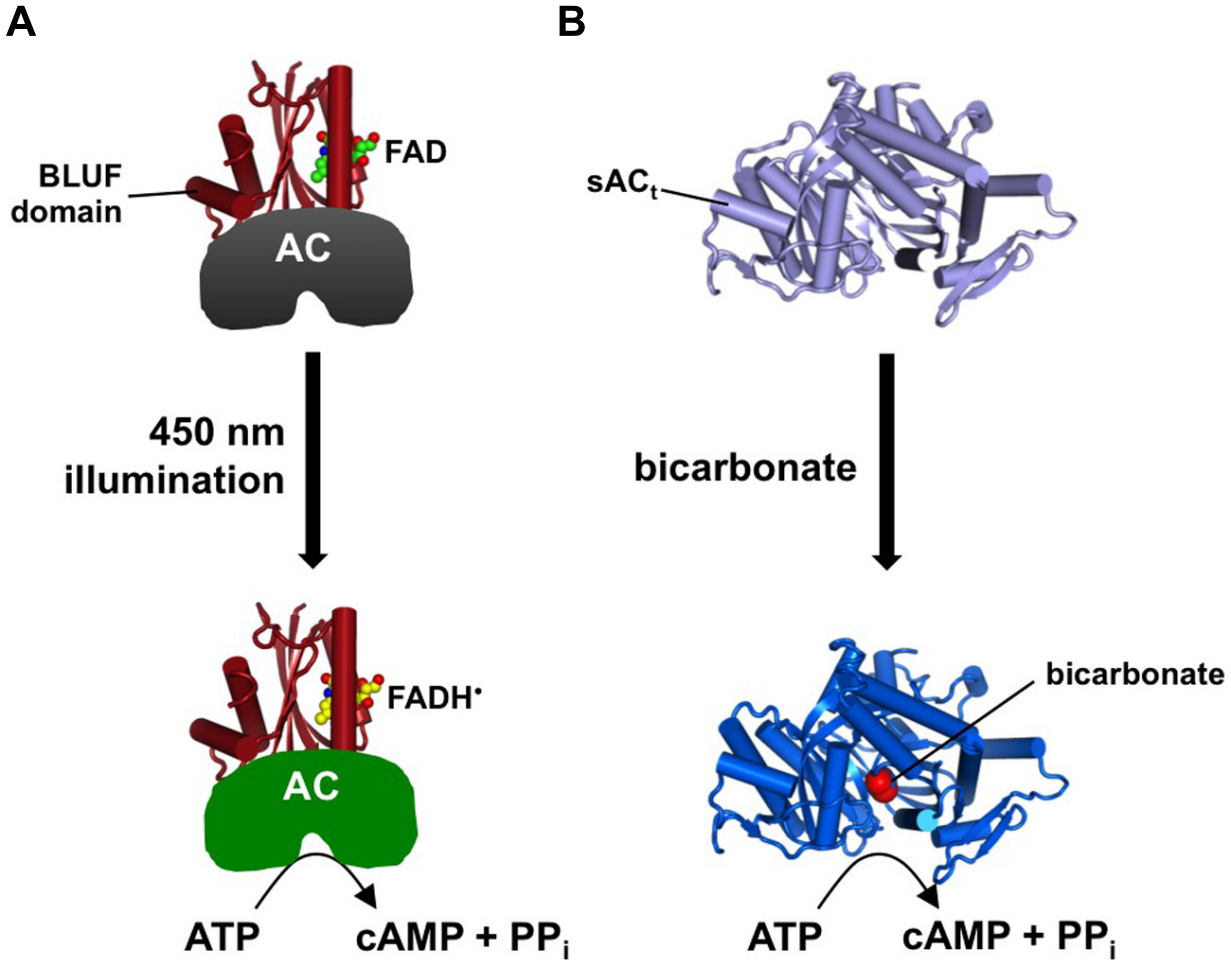
**Tools for triggering cAMP elevation. (A)** Illustration of *Beggiatoa* PAC (bPAC) demonstrating how light illumination of the BLUF domain is coupled to activation of AC activity. **(B)** A truncated version of soluble AC (sAC_t_) responds to elevated bicarbonate by catalyzing cAMP synthesis.

Smaller PACs have subsequently been discovered in species other than *Euglena*, including *Beggiatoa* PAC (bPAC; Ryu et al., 2010; Stierl et al., 2011) (**Figure 2A**). bPAC comprises 350 residues, which facilitates transgenic delivery in comparison to PACα (1019 residues). bPAC also exhibits better responsiveness to blue light than PACα, and cyclase activity decreases faster for bPAC upon return to the dark (Ryu et al., 2010; Stierl et al., 2011). A different class of PAC has been identified in *Microcoleus* (mPAC) that relies on a blue light-responsive light oxygen voltage domain coupled to an AC domain (Raffelberg et al., 2013). mPAC compares favorably to bPAC when expressed *in vivo*, although its responsiveness to blue light is worse *in vitro*, suggesting that a cellular co-factor may support its proper function (Raffelberg et al., 2013). Both bPAC and mPAC require lower light intensity for cyclase activation than PACα (Ryu et al., 2010; Stierl et al., 2011; Raffelberg et al., 2013), so are better suited for application in tissue samples where light penetration is a challenge. A noteworthy alternative to PACs is to employ a C-terminally truncated version of soluble AC (sAC_t_) that can be activated by addition of bicarbonate (Sample et al., 2012) (**Figure 2B**). This approach is compatible with simultaneous monitoring of cAMP concentration and PKA activity with FRET-based probes (Sample et al., 2012; see Combinatorial Applications).

The utility of PACs is demonstrated by studies up to the level of whole animals. PACα has been expressed in cholinergic neurons (Weissenberger et al., 2011), motor neurons (Bucher and Buchner, 2009), and olfactory receptor neurons (ORNs; Bellmann et al., 2010) in *Drosophila*. This has enabled conceptual progress including the establishment of a specific class of ORNs that mediate olfactory avoidance behavior (Bellmann et al., 2010). PACα has been applied to demonstrate a key role for cAMP in growth cone turning in *Xenopus* (Nicol et al., 2011). Nicol et al. (2011) found that local pulses of blue light on distal parts of growth cones expressing PACα were sufficient to maintain orientation of axon outgrowth toward the midline despite blockade of a key Netrin-1 receptor. The potential utility of bPAC has been demonstrated in *Aplysia* (Nagahama et al., 2007), *Toxoplasma* (Hartmann et al., 2013), and in *Xenopus*, HEK293 cells and *Drosophila* (Schroder-Lang et al., 2007). Jansen et al. (2015) have shown how bPAC can be used to control sperm motility and maturation. Furthermore, specific expression of bPAC in *Drosophila* renal tubule in combination with pharmacological manipulations has revealed the distinct roles fulfilled by PKA and Epac in controlling secretion from principal and stellate cells (Efetova et al., 2013). The field is poised for further insights as application of PACs becomes more widespread.

## Sequences for Subcellular Targeting and Modifying Protein–Protein Interactions

An important characteristic of genetically encoding sensors and manipulators of cAMP signaling is that they can be combined with targeting sequences to direct them to specific subcellular compartments. This approach facilitates investigation of spatial aspects of cAMP signaling. In addition, a number of sequences are available for manipulating interaction between PKA and its anchoring sites. A straightforward way to control the location of a given genetically encoded tool is to fuse it with a full-length signaling protein of interest. This option has been taken with proteins including AC8 (Willoughby et al., 2012, 2014; Everett and Cooper, 2013), full-length PKA RII (Zhang et al., 2001), different PDEs (Herget et al., 2008), SOD2 (Zhang et al., 2015), phospholamban (Sprenger et al., 2015), and Hsp20 (Sin et al., 2011). In cases where a good structural understanding is available, fusions are possible to shorter protein domains, such as with the dimerization and docking (D/D) domain of PKA regulatory subunits for tethering at anchoring sites (Di Benedetto et al., 2008), or with the PDE4D-association region from mAKAP (Dodge-Kafka et al., 2005).

The structural interface between PKA and AKAPs is well understood: all AKAPs present a ∼20 amino acid amphipathic helix that binds within a shallow hydrophobic groove on the D/D domain of PKA regulatory subunits (Gold et al., 2006; Kinderman et al., 2006; Sarma et al., 2010). Native and synthetic AKAP anchoring helix sequences are available for modifying PKA anchoring or targeting genetically encoded tools including cAMP sensors (summarized in **Table 1**). A popular sequence is Ht31 (Rosenmund et al., 1994), which is the native anchoring helix of AKAP-Lbc. Using a combination of structural information and peptide array screening, synthetic sequences have been developed with altered binding preferences for RI and RII PKA regulatory subunits. These include the RII-selective sequences AKAP-*is* (Alto et al., 2003), Super-AKAP-*is* (Gold et al., 2006), and AKB-II (Burns-Hamuro et al., 2003); and the RI-selective sequences RIAD (Carlson et al., 2006; Torheim et al., 2009) and AKB-RI (Burns-Hamuro et al., 2003). Studies that utilize sequences for modifying protein–protein interactions typically apply them exogenously as peptides, such as stearated Ht31 (Gold et al., 2012), and delivery approaches in this vein are still improving (Wang et al., 2014, 2015). Some studies have taken advantage of their genetic encodability. For example, sequences including AKAP-*is* (Dodge-Kafka et al., 2005) and AKB-RI/RII (Burns-Hamuro et al., 2003) have been expressed using transfection, and Ht31 has been incorporated in a transgenic mouse (Park et al., 2014).

**Table 1:** Sequences for modifying interactions between cyclic AMP (cAMP) signaling proteins and subcellular targeting.

Purpose	Name and specifications	Reference
**Sequences for modifying protein–protein interactions**		
Binding to the D/D domain of PKA RII subunits	Ht31 (AADLIEEAASRIVDAVIEQVKA). *K*_D_ = 2.2 nM for RIIα; 1.3 μM for RIα	Rosenmund et al. (1994), Alto et al. (2003)
	AKAP-*is* (QIEYLAKQIVDNAIQQA). *K*_D_ = 0.4 nM for RIIα, 277 nM for RIα	Alto et al. (2003), Dodge-Kafka et al. (2005)
	Super-AKAP-*is* (QIEYVAKQIVDYAIHQA). On filter assay binds RIIα 4× more and RIα 12.5× less efficiently than AKAP-*is*	Gold et al. (2006)
	AKB-RII (VQGNTDEAQEELLWKIAKMIVSDVMQQ). *K*_D_ = 2.7 nM for RIIα, 2.5 μM for RIα	Burns-Hamuro et al. (2003)
Binding to the D/D domain of PKA RI subunits	RIAD (LEQYANQLADQIIKEATE). *K*_D_ = 1 nM for RIα, 1800 nM for RIIα	Carlson et al. (2006), Torheim et al. (2009)
	AKB-RI (FEELAWKIAKMIWSDVFQQ). *K*_D_ = 5.2 nM for RIα; 450 nM for RIIα	Burns-Hamuro et al. (2003)
Localizing with type I PKA	RIα (1–64)	Di Benedetto et al. (2008)
Binding to AKAP anchoring helices	RIIβ (amino acids 1–49)	Di Benedetto et al. (2008)
Specifically binding to the AKAP18 anchoring helix	RSelectAKAP18 (PKA RIIα 1–45 with I3V, I5L, T10D, Q14G substitutions)	Gold et al. (2013a)
Binding to PDE4D	mAKAP (1286–1831)	Dodge-Kafka et al. (2005)
**Sequences for subcellular targeting**		
Plasma membrane	C-terminal addition of polybasic-CAAX sequence, e.g., GKKKKKKSKTKCVIM. CAAX box undergoes farnesylation	DiPilato et al. (2004), Dyachok et al. (2006), Saucerman et al. (2006), Depry et al. (2011)
Plasma membrane (cholesterol-rich)	N-terminal addition of Lyn kinase sequence, e.g., MGCIKSKRKDNLNDD, that undergoes myristoylation and palmitoylation	Terrin et al. (2006), Depry et al. (2011), Sample et al. (2012)
Nuclear localization	C-terminal addition of nuclear localization signal PKKKRKVEDA	DiPilato et al. (2004), Terrin et al. (2006), Sample et al. (2012)
Nuclear export	C-terminal addition of nuclear export sequence LPPLERLTL	Sample et al. (2012)
Sarcoplasmic reticulum	C-terminal addition of the helical transmembrane region (PQQARQKLQNLFINFCLILICLLLICIIVMLL) of phospholamban	Liu et al. (2011)
Outer mitochondrial membrane	N-terminal addition of the targeting peptide yTom70	Lefkimmiatis et al. (2013)
	N-terminal addition of MitoDAKAP1 (MAIQLRSLFPLALPGMLALLGWWWFFSRKK), a mitochondrial signal sequence derived from D-AKAP1	Burns-Hamuro et al. (2003), DiPilato et al. (2004), Lim et al. (2007), Depry et al. (2011)
Mitochondrial matrix	N-terminal addition of the mitochondrial matrix targeting signal encoded in the first 12 amino acids of subunit IV of human cytochrome oxidase *c*	DiPilato et al. (2004), Lefkimmiatis et al. (2013)

An additional recent innovation is the development of variants of PKA regulatory subunits with selective anchoring properties (Gold et al., 2013a). A structure-based bacteriophage screening procedure (Walker-Gray and Gold, 2015) enabled identification of variants of the RII D/D domain with substitutions that enable selective anchoring to AKAP18 and AKAP2 (Gold et al., 2013a). RSelect subunits can potentially be applied to drive PKA either specifically to or away from individual AKAPs (Gold et al., 2013a). In most tissues, with rare exceptions (Gold et al., 2012), multiple AKAPs are present and an important challenge is to identify the PKA substrates associated with different AKAP–PKA signaling complexes (Gold et al., 2013b). It is possible to genetically abrogate specific PKA anchoring sites, for example, in the case of AKAP150 ΔPKA knock-in mice in which the PKA anchoring helix in AKAP150 is absent (Murphy et al., 2014). Therefore, a potentially more novel application of RSelect subunits is to combine them with anchoring disruptors such as Ht31. This approach enables PKA catalytic subunits to be driven to a single AKAP within the cell (Gold et al., 2013a).

A variety of sequences may be utilized for targeting to organelles (**Table 1**). Inclusion of C-terminal polybasic-CAAX sequences enables targeting to the plasma membrane (DiPilato et al., 2004; Dyachok et al., 2006; Saucerman et al., 2006; Depry et al., 2011), whereas N-terminal addition of a sequence derived from Lyn kinase targets specifically to cholesterol-rich regions of plasma membrane (Terrin et al., 2006; Depry et al., 2011; Sample et al., 2012). C-terminal addition of the sequence ‘PKKKRKVEDA’ enables nuclear localization (DiPilato et al., 2004; Terrin et al., 2006; Sample et al., 2012), whereas addition of ‘LPPLERLTL’ at the same terminus drives nuclear export (Sample et al., 2012). Targeting to the sarcoplasmic reticulum is possible by fusing with the helical transmembrane domain of phospholamban (Liu et al., 2011). Furthermore, sequences are available for targeting to both the outer mitochondrial membrane (OMM; Burns-Hamuro et al., 2003; DiPilato et al., 2004; Lim et al., 2007; Depry et al., 2011; Lefkimmiatis et al., 2013), and to the mitochondrial matrix (DiPilato et al., 2004; Lefkimmiatis et al., 2013). At the whole animal level, systems are also available for driving cell-type specific expression of genetically encoded tools. These include the UAS/GAL4 system in *Drosophila* (Shafer et al., 2008), and the reverse tetracycline transactivator (rtTA)/doxycycline system in transgenic mice (Kim et al., 2008). Overall, there is an impressive arsenal of sequences at the experimenter’s disposal for manipulating the location and binding properties of cAMP signaling proteins and genetically encoded tools.

## Combinatorial Applications

The tools described in this review have been combined in different ways to make conceptual breakthroughs that would not have been possible if the tools were applied in isolation. The majority of combinations consist of fusions to either sequences derived from cAMP signaling proteins or to subcellular targeting sequences. We will also consider two exceptional studies that have utilized highly innovative combinations of the tool set (Ni et al., 2011; Sample et al., 2012).

The first study involving AKAR1 set a precedent for combining a cAMP sensor with sequences derived from cAMP signaling proteins. Fusion of AKAR1 to the PKA RII subunit revealed that PKA phosphorylation occurs more quickly if PKA is tethered in proximity to its substrate (Zhang et al., 2001). This finding underlined the importance of anchoring PKA to its substrates. A similar approach has been to fuse an Epac-based cAMP sensor to AC8 (Willoughby et al., 2012, 2014), for example, to demonstrate that this Ca^2+^-sensitive AC responds to Ca^2+^ entering through L-type Ca^2+^ channels (Everett and Cooper, 2013). Fusions to isolated domains of cAMP signaling proteins have also been insightful. Fusing Epac-based sensors to the D/D domains of different PKA regulatory subunit classes (Di Benedetto et al., 2008; Roder et al., 2009) has helped to establish that PDE4 associates with RII subunits, whereas PDE2 acts in the vicinity of RI subunits (Di Benedetto et al., 2008). By applying this approach in cardiomyocytes, Di Benedetto et al. (2008) also revealed that the β-AR agonist isoproterenol triggers relatively higher cAMP accumulation with RII subunits, whereas hormones including glucagon induce raised cAMP in the vicinity of the RI subunit. This mechanism allows G protein-coupled receptor specific patterns of cAMP signaling to occur within the same cell. A fusion of the PDE-binding region of mAKAP (residues 1286–1831) with the AKAR2 reporter has also been applied to demonstrate that dominant active MEK5 prolongs PKA activity after cAMP elevation with forskolin (Dodge-Kafka et al., 2005).

Targeting FRET-based sensors using subcellular targeting sequences has also proved to be valuable. Fusions of AKAR reporters with membrane-targeting sequences (Saucerman et al., 2006; Depry et al., 2011) have revealed, for example, that basal PKA activity is higher in cholesterol-rich ‘raft’ regions of membrane (Depry et al., 2011). Similarly, plasma membrane-targeting of an Epac-based cAMP sensor has helped to establish how a pre-assembled protein complex including RXFP1, AKAP79, AC2, β-arrestin 2, and PDE4D3 enables responses to sub-picomolar circulating concentrations of relaxin peptide (Halls and Cooper, 2010). Comparison of PKA activity using AKAR4 targeted to either the mitochondrial matrix or OMM shows that PKA phosphorylation is more enduring at the OMM than in the cytosol due to diminished phosphatase activity at the OMM (Lefkimmiatis et al., 2013). A related approach has been to fuse a sequence that targets to the outer mitochondrial membrane to the PKA regulatory subunit-specific anchoring disruptors AKB-R1 and AKB-R2 (Lim et al., 2007). Lim et al. (2007) expressed these fused sequences to prevent access of either type I or type II PKA to the plasma membrane. This approach revealed that leading-edge phosphorylation of PKA substrates requires type I PKA, with re-localization of type I PKA to the mitochondria inhibiting both the directional persistence and speed of cell migration (Lim et al., 2007).

Two highly innovative combinatorial approaches have been exploited to examine cAMP dynamics in recent years (Terrin et al., 2006; Sample et al., 2012). Sample et al. (2012) developed a novel technique called spatiotemporal manipulation of cAMP using sAC_t_ (Sample et al., 2012). They targeted sAC_t_ by fusing the bicarbonate-activated AC to sequences that target to either the plasma membrane, cytosol, or the nucleus (Sample et al., 2012). By monitoring cAMP accumulation or PKA activity with cytosolic or membrane-tethered sensors, the authors revealed that cAMP and PKA activity can be localized at either the plasma membrane or in the nucleus. Modeling the responses of cAMP and PKA to forskolin and nuclear sAC indicated that a pool of PKA resides in the nucleus. This prediction was subsequently confirmed by immunohistochemistry and immunoblotting (Sample et al., 2012). Another elegant innovation has been to red-shift FRET-based sensors of cAMP and PKA activity to enable simultaneous application with other fluorescent sensors. Red-shifted sensors typically include red fluorescent protein (RFP) variants such that FRET emission occurs at longer wavelengths than with typical fluorescent probes (Ni et al., 2011). This approach was taken to study a cAMP-Ca^2+^-PKA oscillatory circuit in MIN6 cells (Ni et al., 2011). Ni et al. (2011) performed imaging of Fura-2 with either red-shifted AKAR or a red-shifted Epac-based cAMP sensor. Remarkably, the authors also utilized a novel dual detector that enables simultaneous PKA activity and cAMP detection (ICUEPID). Application of these tools showed that oscillations in MIN6 cells can be triggered by cAMP alone, and the authors speculate that such oscillations may provide a way for local PKA activity to be maintained for long periods of time (Ni et al., 2011). These two studies from the Zhang laboratory exemplify the potential benefits of combining different categories of tools in novel ways.

## Considerations for Experimental Design

When deciding whether to employ one of the tools outlined in this review, one considers how the strengths, limitations, and challenges associated with the tool match up with the aims of an experiment. If spatial aspects of cAMP signaling are the emphasis of investigation, then the Epac-based cAMP probes (Klarenbeek et al., 2011; Li et al., 2015) are a good option as they consist of relatively short sequences that can be directed to subcellular locations with targeting sequences (**Table 1**). On the other hand, if a maxim is placed on temporal resolution, the CNG2 system may be the better option (Rich et al., 2000; Fagan et al., 2001). Similarly, PACs such as bPAC (Ryu et al., 2010; Stierl et al., 2011) allow faster activation and de-activation than the bicarbonate-activated sAC_t_ (Sample et al., 2012). For tandem applications, the bicarbonate-activated sAC_t_ cyclase has the advantage that there is no concern about unwanted photoactivation when combined with FRET-based cAMP/PKA sensors (Sample et al., 2012). Similarly, red-shifted sensors can allow cAMP and PKA activity sensors to be applied simultaneously (Ni et al., 2011). The choice of tools will also be dictated by the availability of specialist equipment. For example, application of FRET-based sensors typically relies on a confocal microscope with appropriate lasers. If such a microscope is unavailable then recordings may be performed using a plate reader (Robinson et al., 2014) although studies will be limited to the population level following this approach.

It is important to consider the potential off-target effects and distortions that may be caused by the tools. For example, if PKA is the focus of investigation, it is wise to avoid PKA-based cAMP sensors that may distort signaling by interacting with native PKA subunits. Any unwanted cellular changes resulting from long-term expression of genetically encoded tools can be limited by using inducible expression systems such as the tetracycline system (Meyer-Ficca et al., 2004). Another consideration is that cAMP sensors act as buffers for cAMP, potentially altering the amplitude and duration of cAMP transients in a similar way to alteration of free Ca^2+^ transients by dyes such as Fura-2 (Neher, 2008). Buffering effects can be at least ameliorated by taking care to express cAMP sensors at levels no higher than is necessary for reliable detection. Finally, one should also bear in mind that targeting and disruptor sequences may not always behave as desired. The specificity of disruptor sequences can be tested by performing negative control experiments with sequences such as Ht31-P in which the disruptor helix is destabilized by incorporation of a central proline (Willoughby et al., 2006). It is also good practice to image cells to check that localization sequences have partitioned within the cell as anticipated (Lim et al., 2007). Overall, the wide range of available tools means that a good technical solution is at hand for most experimental aims.

## Conclusions and Prospects

The genetically encoded tool set for investigating cAMP signaling has expanded rapidly over the last 15 years. There are now a multitude of options for monitoring intracellular fluctuations in cAMP, with Epac-based probes emerging as the most popular sensor class (DiPilato and Zhang, 2009; Klarenbeek et al., 2011). AKAR reporters enable PKA activity to be monitored more directly, while experimenters can artificially elevate cAMP levels by photoactivating PACs or stimulating sAC with bicarbonate. The functionality of these tools can be enhanced by combining with an impressive array of sequences for modifying protein–protein interactions and subcellular targeting (**Table 1**).

There is scope for improving the tools described in this review, developing novel tools, and combining the tools in new ways. Biosensors typically improve over time by incorporating modifications based on user feedback (Oldach and Zhang, 2014). This process may have reached the point of diminishing returns for FRET-based sensors and peptides derived from AKAP anchoring helices, whereas there is more potential for improvement of the more recently developed PACs and RSelect subunits. For example, an AC with a synthetic domain architecture has been shown to be activated by near-infrared light (Ryu et al., 2014). This PAC is likely to be particularly advantageous for studies that require deep tissue penetration (Ryu et al., 2014). It is worth noting that methods for calibrating FRET-based cAMP reporters are still improving (Koschinski and Zaccolo, 2015). Genetically encoded sensors that incorporate Renilla luciferase for Bioluminescence resonance energy transfer (BRET) imaging (Prinz et al., 2006; Jiang et al., 2007) exhibit higher maximal signal-to-noise ratios than FRET-based sensors. Application of BRET sensors in live cell imaging could become more popular if light detectors can improve to overcome the relatively low light output of BRET compared to FRET. Although sequences for disrupting or selectively binding to either side of the AKAP–PKA interface are advanced (**Table 1**), no equivalent sequences exist for interactions mediated by ACs, PDEs, or Epacs. If the structural and molecular basis of interactions involving these protein classes can be determined more precisely, it should be relatively straightforward to develop improved sequences using established peptide array screening (Alto et al., 2003; Burns-Hamuro et al., 2003) and directed evolution (Walker-Gray and Gold, 2015) approaches.

There are currently no existing technologies for activating PKA or Epac activity with temporal precision. One future avenue is to genetically encode unnatural amino acids such as caged lysine using the amber codon TAG (Gautier et al., 2011; Kim et al., 2013) as a basis for enzyme activation upon illumination in a similar way to PACs. Future studies may also exploit an approach for light-gating protein-protein interactions that can be genetically encoded using sequences from a phytochrome signaling network (Levskaya et al., 2009). Further combinatorial possibilities that have been underexploited include fusions of PACs and sAC_t_ with the sequences listed in **Table 1**. Many targeting studies have focused on the relation of cAMP signaling with different sub-structural features of cardiomyocytes (Di Benedetto et al., 2008; Nikolaev et al., 2010). It will be valuable to perform analogous experiments to probe cAMP signaling in different neuronal compartments. Finally, the ICUEPID sensor (Ni et al., 2011) sets a precedent for how red-shifting FRET-based sensors can enable their combination with other technologies that rely upon illumination.

It is important to question the value of determining how cAMP signaling processes proceed with high spatial and temporal detail. The conceptual breakthrough that supported the development of the first β-blocker propranolol (Stapleton and Black, 1997) indicates how current research targeted at spatiotemporal cAMP signaling might be useful in the long term. James Black was inspired to target β-ARs by experiments conducted by Raymond Ahlquist. Ahlquist established that there are two major classes of adrenergic receptor, with β-ARs underpinning responses to norepinephrine in the heart (Stapleton and Black, 1997). An analogous classification of cAMP signaling processes at the sub-cellular level can provide a framework for selectively intervening in cAMP signaling with more precision than drugs that act at the level of the receptor. Combinatorial application of the genetically encoded tools described in this review is central to achieving the level of spatiotemporal detail necessary to open a path to a next generation of drugs that manipulate cAMP signaling.

## Conflict of Interest Statement

The authors declare that the research was conducted in the absence of any commercial or financial relationships that could be construed as a potential conflict of interest.
